# Analysing the potential of hydrophilic adhesive systems to optimise orthodontic bracket rebonding

**DOI:** 10.1186/s13005-020-00233-3

**Published:** 2020-09-05

**Authors:** Isabel Knaup, Antonia Böddeker, Katrin Tempel, Eva Weber, Jenny Rosa Bartz, Marcia Viviane Rückbeil, Rogério Bastos Craveiro, Yvonne Wagner, Michael Wolf

**Affiliations:** 1 Department of Orthodontics, RWTH Aachen University Hospital, Pauwelsstr. 30, 52074 Aachen, Germany; 2 Department of Medical Statistics, RWTH Aachen University Hospital, Pauwelsstr. 19, 52074 Aachen, Germany; 3 Department of Orthodontics, Jena University Hospital, An der alten Post 4, 07743 Jena, Germany

**Keywords:** Rebonding, Second bonding, Orthodontics, Bracket, Shear bond strength

## Abstract

**Introduction:**

Bond failure during fixed orthodontic treatment is a frequently occurring problem. As bracket rebonding is associated with reduced shear bond strength, the aim of the present investigation is to analyse the effect of different innovative rebonding systems to identify optimised rebonding protocols for orthodontic patient care.

**Methods:**

Metallic brackets were bonded to the frontal enamel surfaces of 240 bovine lower incisors embedded in resin bases. Teeth were randomly divided into two major experimental groups: in group 1 a hydrophilic primer (Assure™ PLUS) was compared to commonly used orthodontic adhesives (Transbond XT™, BrackFix®, Grengloo™) and a zero control. In group 2 different rebonding systems were analysed using a hydrophilic primer (Assure™ PLUS), a methyl methacrylate-consisting primer (Plastic Conditioner) and a conventional adhesive (Transbond XT™). All teeth were tested for shear bond strength according to the DIN-13990 standard, the Adhesive Remnant Index and enamel fracture rate.

**Results:**

The hydrophilic primer enhanced shear bond strength at first bonding (Assure™ PLUS 20.29 ± 4.95 MPa vs. Transbond XT™ 18.45 ± 2.57 MPa; BrackFix® 17 ± 5.2 MPa; Grengloo™ 19.08 ± 3.19 MPa; Meron 8.7 ± 3.9 MPa) and second bonding (Assure™ PLUS 16.76 ± 3.71 MPa vs. Transbond XT™ 13.06 ± 3.19 MPa). Using Plastic Conditioner did not seem to improve shear bond strength at rebonding (13.57 ± 2.94). When enamel etching was left out, required shear bond strength could not be achieved (Plastic Conditioner + Assure™ PLUS 8.12 ± 3.34 MPa; Plastic Conditioner: 3.7 ± 1.95 MPa). Hydrophilic priming systems showed decreased ARI-scores (second bonding: 2.63) and increased enamel fracture rates (first bonding: 55%; second bonding 21,05%).

**Conclusions:**

Based on the present study we found that rebonding strength could be compensated by the use of hydrophilic priming systems. The additional use of a methyl methacrylate-consisting primer does not seem to enhance shear bond strength. No etching approaches resulted in non-sufficient bond strength.

## Introduction

Bond failure during fixed orthodontic treatment is an unpredictable problem that is usually the result either of the patient’s accidentally applying inappropriate forces to the bracket or of a poor bonding strength [1]. Bonding performance depends on factors such as tooth type and position, type of bonding agents and curing methods, bracket types and materials, aging and attrition [2, 3]. Prevalence varies between 2.8–12.3%, whereas bracket failure of up to 2 brackets seems to be clinically acceptable [2, 4–7]. Because molars and second premolars are more often affected by bracket failure, some authors recommend bands for these teeth, but as bands affect esthetics and oral hygiene, their use is limited [2, 8–12]. Lower teeth are more affected by bracket loss than upper teeth since saliva contamination during bracket positioning is more likely [3, 13–15].

Since premature bracket loss can lead to unwanted tooth movement and prolonged treatment duration, immediate rebonding is required [16, 17]. Different rebonding procedures are described: clinicians either re-use the failed bracket or they bond a new bracket. For bracket recycling, the remaining adhesive has to be removed from the bracket in either mechanic, thermic or chemical procedures using silicon polishers, sand blasting, flame-scarfing or acid treatment [18, 19]. Remaining adhesive on the teeth is usually removed with slow rotating hard metal polishers, although micro damages remain a frequently observed phenomenon [20]. Concerning shear bond strength (SBS), literature shows heterogeneous results: some studies reported reduced SBS for rebonded or recycled brackets [1, 21–23], but others cannot confirm these findings [24]. However, authors assume that lower bond strength may be related to changes in the morphologic characteristics of the etched enamel surface as a result of the presence of adhesive remnants [1, 24].

To improve the rebonding capability, recently the adhesive systems Assure™ PLUS (AS, Reliance® Orthodontic Products, Inc., Itasca, USA) and Plastic Conditioner (PC, Reliance® Orthodontic Products, Inc., Itasca, USA) with SBS enhancing characteristics have been introduced and might be a promising tool in the improvement of bracket rebonding. Assure™ PLUS consists like other SBS enhancing products of the molecule hydroxyl ethyl methacrylate (HEMA) which is thought to assist with moisture control at bonding [25]. HEMA is initially known for the use in dentin adhesion because its hydrophilic properties and hydrophobic functional groups allow a better penetration and infiltration of monomers resulting in a better SBS after polymerisation [26–28]. Therefore, HEMA-consisting primers are also called hydrophilic primers. Plastic Conditioner consists of the molecules methyl methacrylate (MMA) and isobutyl methacrylate and is claimed to enhance the connection between composite and acrylic resin. In prosthodontics, MMA has been used for repairing prostheses, but it has also become popular in orthodontics for indirect bonding or bracket placement on acrylic teeth [29–32]. However, systematic information on rebonding might improve rebonding strength. Moreover, the effectiveness of additional enamel etching procedure has been questioned. It remains unclear, whether the use of those reagents alone or in combination might improve rebonding. Therefore, the aim of the present investigation was to evaluate the impact of different rebonding procedures and products on the SBS of orthodontic attachments in second bonding.

## Materials und methods

### Trial design

In this in vitro study metallic upper central incisor brackets with laser structured bases (Discovery®, DENTAURUM GmbH & Co. KG, Ispringen, Germany) were bonded to the frontal enamel surfaces of commercially available bovine lower incisors (Rocholl GmbH, Aglasterhausen, Germany) to test for shear bond strength (SBS) according to the DIN-13990 standard. All teeth were caries-free and didn’t show any signs of fractures or breaks. They were stored in pure water of quality 3 (Ampuwa®, Fresenius Kabi Deutschland GmbH, Bad Homburg vor der Höhe, Germany) at 4 ± 2 °C according to DIN ISO-3696 standard and removed shortly before the trial. Teeth were randomly divided into groups, cleaned (SuperPolish™, Kerr Dental, Rastatt, Germany), rinsed and dried for further processing. Sample size was set to 20 specimens per group in accordance to the DIN-13990 standard that requires a minimum of 10 specimens and previous in vitro studies investigating shear bond strength [16, 33]. The trial consisted of three parts.

### Conventional orthodontic bonding systems

In part 1 commonly used light-curing adhesives were tested to assess reference values. Therefore, brackets were bonded on bovine teeth in the following manner: 35% phosphoric acid (Vococid®, VOCO® GmbH, Cuxhaven, Germany) was applied for 20 s, etching agent was removed using water spray, enamel surface was dried and coated with a primer, the bracket was placed using an adhesive paste, surpluses were removed, light curing was performed using a light-emitting diode (LED) device with a light intensity of 3000 mW/cm^2^ (Mini LED™, Acteon Germany GmbH, Düsseldorf, Germany). Therefore, the device was angled at 30 degrees and placed as close as possible to the bracket from each the mesial and distal side for 10 s. Further specimens were bonded with a self-drying glass ionomer luting cement serving as a zero control.

The following groups were built:

- Transbond™ XT primer + adhesive (TB XT, 3 M Unitek GmbH, Neuss, Germany) (*n* = 20)
- BrackFix® primer + adhesive (VOCO® GmbH, Cuxhaven, Germany) (*n* = 20)
- Ortho Solo™ primer + Grengloo™ adhesive (Ormco Corp., Orange, USA) (*n* = 20)
- Meron glas ionomer luting cement (VOCO® GmbH, Cuxhaven, Germany) (*n* = 20)

### Strength enhancing orthodontic bonding systems

In part 2 brackets were bonded in the same manner, but the primer Assure™ PLUS (AS, Reliance® Orthodontic Products, Inc., Itasca, USA) was used instead of the TB XT primer. AS was applied in a thin layer for 10 s and carefully air dried prior to bracket placing with TB XT adhesive.

In addition, part 2 was intended to investigate on the effects of AS on bracket rebonding. Therefore, premature bracket loss was simulated as followed: brackets were bonded with TB XT as described above and debonding was performed with a universal testing machine (Z020, ZwickRoell GmbH & Co. KG, Germany). The remaining adhesive was removed from the teeth with a hard metal polisher (Hager & Meisinger GmbH, Neuss, Germany) and new orthodontic attachments were placed on the same bovine teeth with either TB XT primer or AS primer and TB XT adhesive.

The following groups were built:

- AS + TB XT adhesive (*n* = 20)
- AS + TB XT adhesive (*n* = 20) *rebonding*
- TB XT primer + adhesive (*n* = 20) *rebonding*

### Comparison of different rebonding systems

In part 3 different rebonding procedures and product combinations including a methacrylate-consisting primer (Plastic Conditioner = PC, Reliance® Orthodontic Products, Inc., Itasca, USA) were compared. PC was applied in a thin layer before a primer was applied after 60 s. In some specimens second bonding was performed without applying phosphoric acid.

The following groups were built:

- TB XT primer + TB XT adhesive (*n* = 20) *rebonding*
- PC + TB XT primer + TB XT adhesive (*n* = 20) *rebonding*
- PC + AS + TB XT adhesive (*n* = 20) *rebonding*

*Leaving out enamel etching*

- PC, AS + TB XT adhesive (*n* = 20) *rebonding*
- PC, TB primer + TB XT adhesive (*n* = 20) *rebonding*

### Debonding according to the DIN-13990 standard

**Fig. 1:**
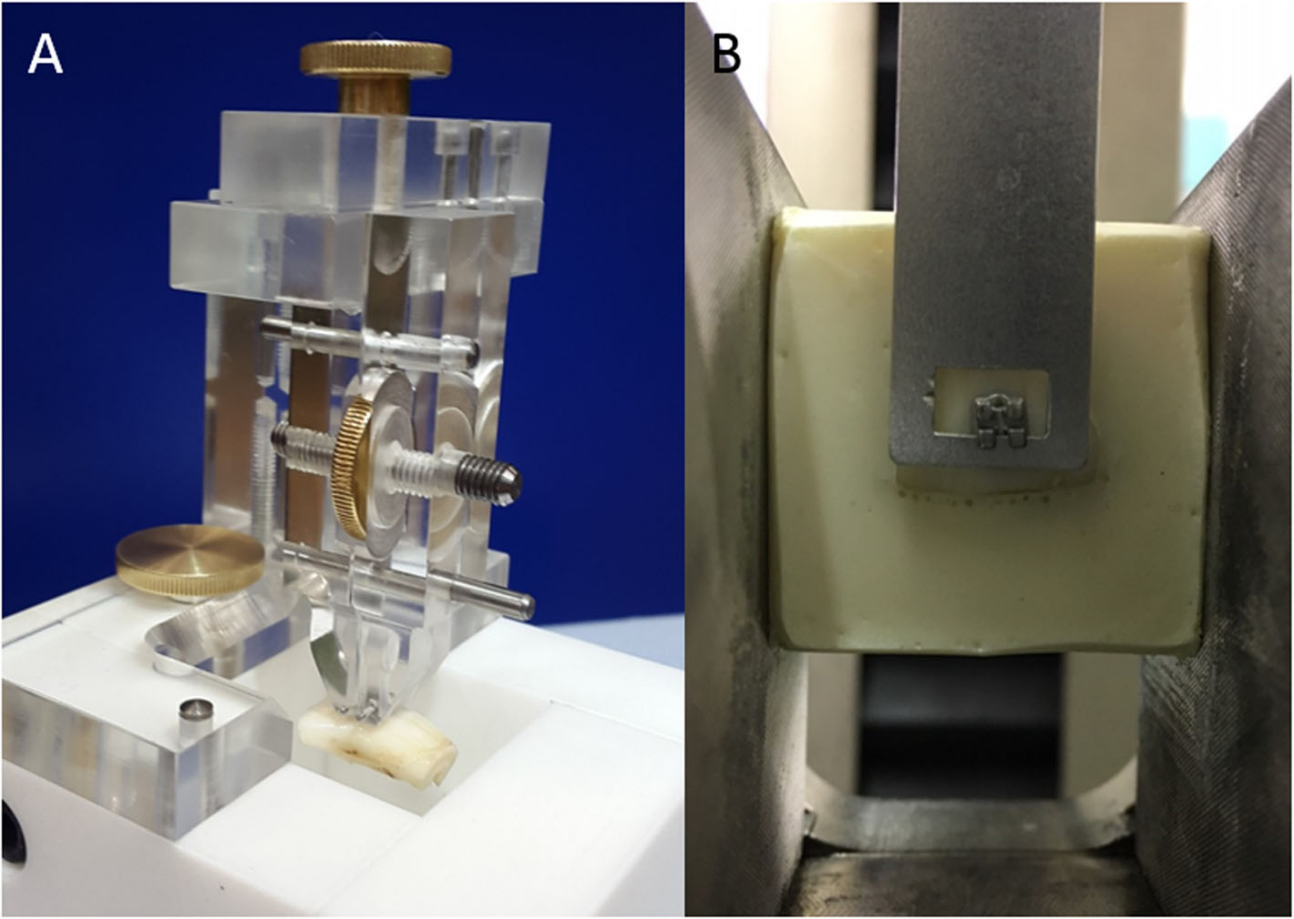
Processing of the specimens. **a** Orthodontic attachments were bonded to lower bovine incisors and embedded in polyurethane in custom-made polytetrafluorethylene devices. **b** After polymerisation devices were unscrewed and the squared specimens were referred to the universal testing machine Zwick

The specimens of all three parts were embedded in polyurethane (CEM 9000, Cloeren Technology GmbH, Wegberg, Germany) in custom-made squared polytetrafluorethylene devices (Otto Schott Institute of Materials Research, University of Jena, Germany) to allow later placement in a universal testing machine (Fig. 1a). Each specimen was processed shortly before testing, stored in water at 37(±2)°C for 24 (±2) h after polymerisation and mounted into a universal testing machine (Z020, ZwickRoell GmbH & Co. KG, Ulm, Germany) (Fig. 1b). The movement of the head and the magnitude of the applied force was electronically controlled and monitored via the software testXpert® II (ZwickRoell GmbH & Co. KG, Ulm, Germany). Prior to testing, 20 specimens were debonded to verify the procedure. The following parameters were recorded according to DIN-13990 standard [34]:

1. Shear bond strength (SBS): the bond strength was measured in shear mode at a crosshead speed of 1 mm/min until bracket removal was achieved. The SBS needed to debond or initiate bracket fracture was recorded and then converted into pressure (MPa) as a ratio of force per bracket surface area (13.12 mm^2^).
2. Adhesive remnant index (ARI): after bracket removal, the enamel surfaces were examined using a light microscope and the corresponding ARI assessed. The following 5-point scale classification based on Bishara and Trulove [35] was used: 1 = no adherence of composite on the bracket base, 2 = less than 10% of composite remaining on the bracket surface, 3 = more than 10% but less than 90% of composite remaining on the bracket surface, 4 = more than 90% of composite remaining on the bracket surface, and 5 = all composite remaining on the bracket base.
3. Enamel fractures: after bracket removal, the enamel surfaces were examined using a light microscope and enamel fractures were scored using 10x magnification. ARI and enamel fracture were scored by one investigator who was not aware of the groups.

### Statistical analysis

Data were recorded in Microsoft Excel files (Office version 365, Microsoft Corporation, Redmond, USA) and transferred to SAS software (PROC MIXED and PROC LOGISTIC, version 9.4, SAS Institute, Cary USA) for analysis. Methodical outliers were excluded. Graphics were created using GraphPad Prism (version 7, GraphPad software, San Diego, USA).

Continuous data are shown as mean ± standard deviation (SD), categorical outcomes as absolute and relative frequencies (%). The outcomes SBS and ARI score were compared using a linear regression model, the outcome enamel damage using a logistic regression model with Firth’s bias correction. Depending on the comparison, the models included either one independent factor or two independent factors and an interaction term. All relevant pairwise comparisons were tested using linear contrasts (lsmeans or lsmestimate option). To account for multiple comparisons, all *p*-values were adjusted using the simulation based adjustment by Edwards and Berry (with EPS = 5∙10^− 4^ and ACC = 2.5∙10^− 4^) [36]. A *p*-value ≤0.05 was used to indicate statistically significant differences.

## Results

### Bonding strength of analysed orthodontic bonding systems

**Fig. 2:**
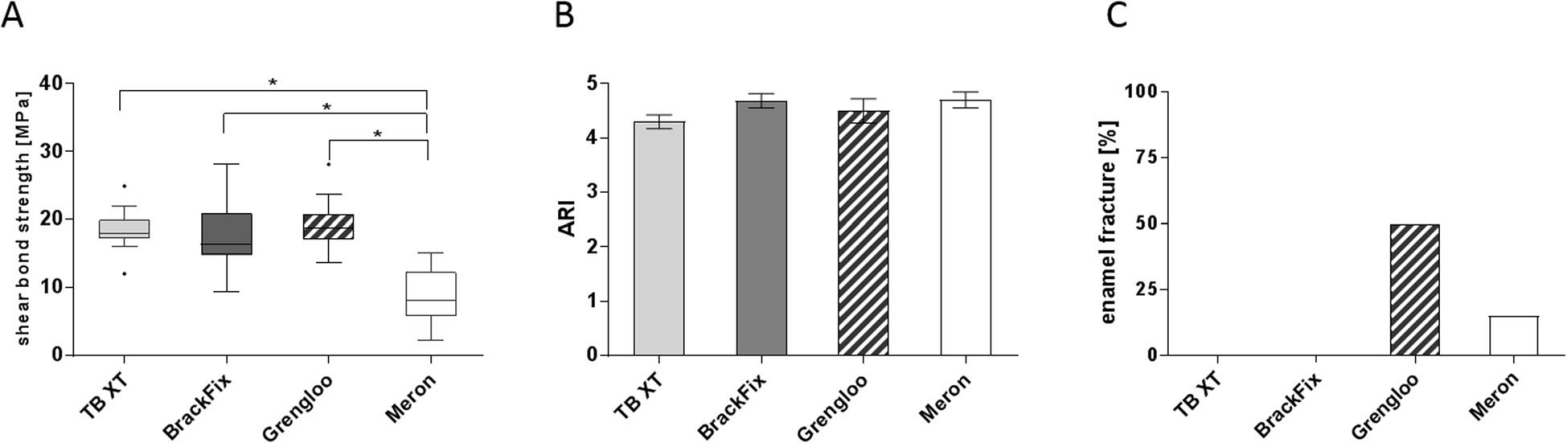
Comparison of conventional orthodontic bonding systems. **a** Shear bond strength, **b** ARI and **c** rate of enamel fractures of orthodontic attachments bonded to bovine incisors with Transbond XT™ (TB XT, *n* = 20), BrackFix® (*n* = 20), Grengloo™ (*n* = 20) and the glass ionomer luting cement Meron (*n* = 20). **a** Shear bond strength of TB XT, BrackFix® and Grengloo™ were in a clinically adequate range, whereas Meron served as a zero control. **b** Mean ARI-scores were between 4 and 5. **c** Enamel fractures were observed in Grengloo™ (50%) and Meron (15%). Data shown as mean ± SEM or as relative frequency; statistically significant differences (*p* ≤ 0.05) are marked with *

Shear bond strength (SBS) of TB XT (18.45 ± 2.56 MPa, *gold standard*), BrackFix® (17 ± 5.2 MPa) and Grengloo™ (19.08 ± 3.19 MPa) was in a clinically adequate range without significant differences. All three products showed significantly higher mean SBS than glass ionomer luting cement (8.7 ± 3.86 MPa, *zero control*) (Fig. 2a). Mean ARI-scores ranged between 4 and 5 in all four products (Fig. 2b), whereas the rate of enamel fractures was higher in Grengloo™ (50%) and Meron (15%) than in TB XT and BrackFix® (Fig. 2c). Table 1 shows the results for the main effects of comparisons.

**Table 1:** Results for the main effects of comparisons 1-3

Comparison	Main effect(s)	SBS *p*-value ^a^	ARI *p*-value ^a^	Enamel fractures *p*-value ^b^	Pairwise comparisons
Part 1 - *Conventional orthodontic bonding systems*	Group (4 levels)	< 0.0001	0.2734	0.0054	Statistically significant differences are indicated in Fig. 2a-c.
Part 2 - *Strength enhancing orthodontic bonding systems*	Primer (2 levels)	0.0014	< 0.0001	0.0046	Statistically significant differences are indicated in Fig. 3a-c.
	Rebonding (2 levels)	< 0.0001	0.0043	0.8798	
	Primer*Rebonding	0.2675	0.0003	0.1629	
Part 3 - *Comparison of different rebonding systems*	Group (7 Levels)	< 0.0001	< 0.0001	0.0008	Statistically significant differences are indicated in Fig. 4a-c.

^a^ The *p*-values for the main effects were obtained from a linear regression model

^b^ The *p*-values for the main effects were obtained from a logistic regression model with Firth’s bias correction.

### Effect of strength enhancing orthodontic bonding systems and rebonding on bonding quality

**Fig. 3:**
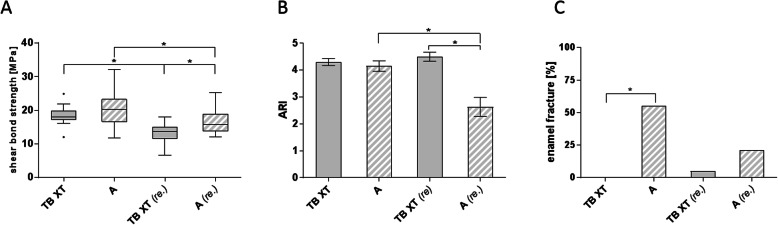
Comparison of hydrophilic bonding systems. **a** Shear bond strength, **b** ARI and **c** rate of enamel fractures of orthodontic attachments bonded to bovine incisors with Transbond XT™ (TB XT) or Assure™ PLUS primer and TB XT adhesive at first bonding and at rebonding after artificial bracket loss (each *n* = 20). **a** Assure™ PLUS enhanced shear bond strength at first bonding and at rebonding, whereas rebonding showed in general lower values. **b** Mean ARI-score was significantly lower at rebonding with Assure™ PLUS. **c** The rate of enamel fractures at first bonding was significantly higher in Assure™ PLUS than in TB XT. A = Assure™ PLUS; re. = rebonding. Data shown as mean ± SEM or as relative frequency; statistically significant differences (*p* ≤ 0.05) are marked with *

When A primer was combined with TB XT, SBS of orthodontic brackets increased compared to TB XT primer and adhesive alone (20.29 ± 4.95 vs. 18.45 ± 2.56 MPa), but the difference was not statistically significant (Fig. 3a). In general, rebonding decreased the mean SBS significantly in both AS primer and TB XT adhesive (16.76 ± 3.7 MPa) and TB XT primer and adhesive alone (13.06 ± 3.19 MPa) compared to first bonding. Mean SBS at second bonding was significantly higher in AS primer and TB XT adhesive compared to TB XT alone.

At first bonding, mean ARI-scores of TB XT and AS ranged between 4 and 5. At second bonding, TB XT showed the same range, while AS and TB XT adhesive showed significantly lower ARI-Scores (2.63 ± 1.5) suggesting a higher risk for enamel damage (Fig. 3b). In general, the rate of enamel fractures was higher in AS (first bonding: 55%, rebonding: 21.05%) than in TB XT (first bonding: 0%, second bonding: 5%), while the difference was significant only at first bonding (Fig. 3c).

### Comparison of different rebonding systems

**Fig. 4:**
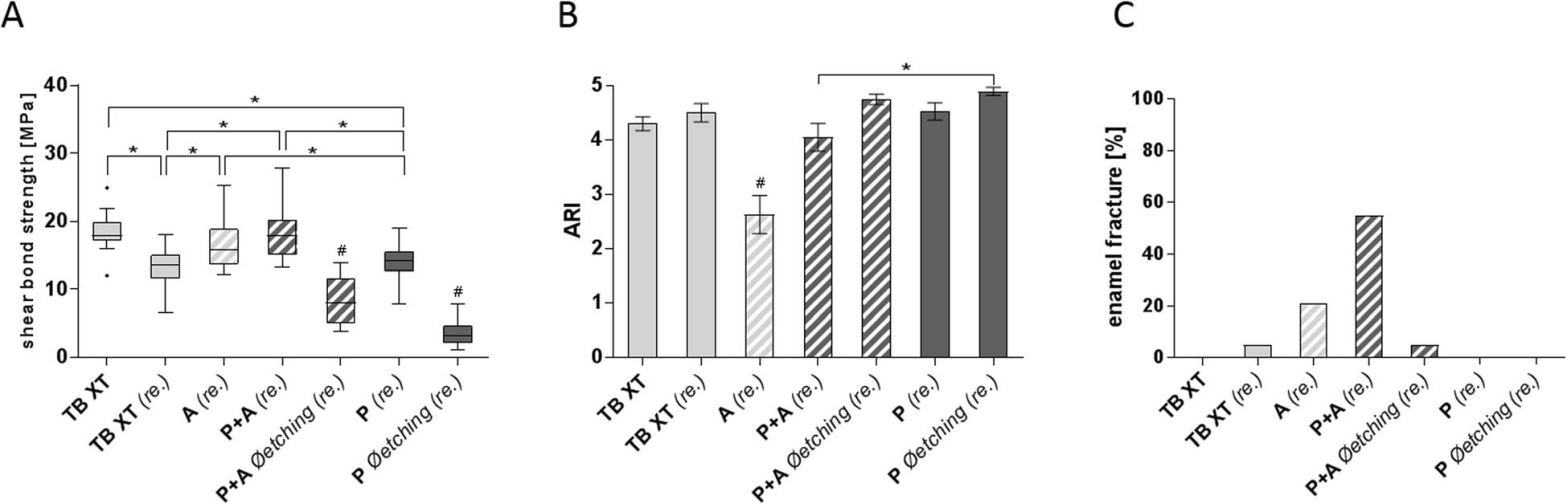
Comparison of different rebonding systems. **a** Shear bond strength, **b** ARI and **c** rate of enamel fractures of orthodontic attachments bonded to bovine incisors with Transbond XT™ (TB XT) after artificial bracket loss. Different primers and product combinations were used with or without enamel etching (each *n* = 20). **a** Rebonding led to a decreased shear bond strength, whereas a low shear bond strength could be compensated by the use of Assure™ PLUS. No etching approaches resulted in non-sufficient SBS. Plastic Conditioner did not seem to further enhance shear bond strength at rebonding. **b** Mean ARI-score ranged between 4 and 5, but significant lower in Assure™ PLUS (2,63 ± 1,54). **c** The rate of enamel fractures was higher in Assure™ PLUS. A = Assure™ PLUS, P=Plastic Conditioner, re. = rebonding, Øetching = no usage of phosphoric acid. Data shown as mean ± SEM or as relative frequency; statistically significant differences (*p* ≤ 0.05) are marked with * and # (significant differences to all other groups)

Processing of bovine teeth with AS (16.76 ± 3.70 MPa) or AS in combination with PC (18.30 ± 3.99 MPa) prior to rebonding led to significantly increased SBS compared to applying TB XT alone (Fig. 4a). There were no statistically significant differences to the gold standard (18.45 ± 2.56 MPa).

The use of PC, TB XT primer and adhesive did not show significant differences to rebonding with TB XT primer and adhesive alone (13.57 ± 2.94 vs. 13.06 ± 3.19 MPa). Leaving out enamel etching led to clinically inadequate SBS in the rebonding procedures with PC and AS (8.12 ± 3.34 MPa) as well as PC, TB XT primer and adhesive (3.7 ± 1.95 MPa). Mean ARI-score ranged between 4 and 5, but was significantly lower in Assure™ PLUS (2.63 ± 1.54) (Fig. 4b). Concerning enamel fractures, there was a higher rate of fractures at rebonding in AS (21.05%) and in AS in combination with PC (55%). Enamel fractures in other combinations were absent or the rate was low (TB XT, PC leaving out etching = 5%) (Fig. 4c).

## Discussion

The present in vitro study aims to evaluate the effect of different bonding and rebonding procedures on shear bond strength (SBS) of orthodontic attachments, adhesive adherence and enamel fractures in rebonding conditions. As orthodontic attachments usually remain in the patient’s mouth for about 2 years, adequate bonding strength should not be below 6–10 MPa [37, 38]. Moreover, these values are also required for a permanent retention with fixed orthodontic retainers [39, 40].

In our investigation, initial bonding of attachments was performed with TB XT, BrackFix® and Grengloo™. SBS ranged between 16–18 MPa and can be compared to similar studies [41]. Hence, the clinical relevance is therefore given. The use of AS further increased SBS and also led to a higher enamel fracture rate. So the use of AS at first bonding might remain reserved for complicated bonding situations such as porcelain restorations or saliva contamination.

Rebonding was performed with the adhesive system TB XT in different primer combinations. When rebonding was performed with TB XT alone, SBS decreased for about 5 MPa on overage compared to the *gold standard* (13.06 ± 3.19 vs. 18.45 ± 2.56 MPa). Previous authors confirm our findings as they found lower SBS in rebonding than in initial bonding [1, 21, 24, 42].

If acid etching was not performed at all, SBS decreased for about 10 MPa when AS and PC primer were applied (8.12 MPa) and for about 15 MPa when PC and TB XT primer were applied (3.7 MPa). Therefore, the use of the primers PC and TB XT alone cannot be recommended in rebonding because the achieved values were lower than proposed by Reynolds [37].

When AS or AS and PC were used in rebonding, differences in SBS to initial bonding were compensated and the gold standard was reached. However, many studies have shown the benefit of AS in enhancing SBS in initial bonding in ceramic and metallic restorations, but to our knowledge, the impact on rebonding remained unclear [43–46]. Since a higher SBS at rebonding also led to higher rates of enamel fractures in our investigation, bracket rebonding should be done with caution in cases of hydrophilic primers. In vitro studies showed that enamel loss ranged between 7.6-41,6 μm resulting from debonding molar tubes and 18-33 μm from debonding ceramic brackets [47, 48].

Rüger et al. reported in general a higher risk of enamel tear-outs during debonding at repeated etching, but also showed increased SBS when acid etching was left off (4.95 ± 1.22 MPa) [49]. The authors postulate, that acid etching could be left off in rebonding, when composite was reduced instead of completely removed [49]. Our results also show, that SBS was reduced, when acid etching was left out and SBS was only clinically adequate, when teeth were processed with AS. However, leaving out acid etching also led to a reduced rate of enamel fractures.

Concerning the application of PC in rebonding procedures, there is only limited evidence. Egan et al. investigated different rebonding procedures in human teeth and applied a combination of PC and Enhance™ Adhesion Booster. However, likewise our investigation they didn’t find increased SBS [50].

### Limitations

With respect to the findings of this investigation, there are a few limitations to note. First, results should be interpreted with caution as they were of theoretical character and reflect an in vitro approach. It has to be considered, that SBS measured at debonding in vitro is on average 40–57% higher than measured in vivo [51, 52]*.* Reasons might be material aging and unpredictable temperature changes in the oral cavity, which can alter material properties and cannot be fully transferred to in vitro approaches [53]. Second, we used bovine incisors in our study and therefore, the results may not be fully transferable to human teeth. Nevertheless, the results of this study can serve as a basis for further prospective research in order to enable reliable assessments and clinical implications about rebonding procedures*.*

## Conclusion

- Based on the present findings limited rebonding strength can be compensated by the use of hydrophilic priming systems.
- The additional use of plastic conditioners does not seem to enhance bonding strength.
- No etching approaches resulted in non-sufficient bonding strength.

## Data Availability

The datasets used and/or analysed during the current study are available from the corresponding author on reasonable request.

## Abbreviations

SBS: Shear bond strength
ARI: Adhesive remnant index
TB XT: Transbond™ XT
AS: Assure™ PLUS
PC: Plastic Conditioner
